# Attenuated AMPA Receptor Expression Allows Glioblastoma Cell Survival in Glutamate-Rich Environment

**DOI:** 10.1371/journal.pone.0005953

**Published:** 2009-06-18

**Authors:** Dannis G. van Vuurden, Maryam Yazdani, Ingeborg Bosma, Aart J. F. Broekhuizen, Tjeerd J. Postma, Jan J. Heimans, Paul van der Valk, Eleonora Aronica, Bakhos A. Tannous, Thomas Würdinger, Gertjan J. L. Kaspers, Jacqueline Cloos

**Affiliations:** 1 Department of Pediatric Oncology/Hematology, VU University Medical Center, Amsterdam, the Netherlands; 2 Neuro-oncology Research Group, Cancer Center Amsterdam, VU University Medical Center, Amsterdam, the Netherlands; 3 Department of Neurology, VU University Medical Center, Amsterdam, the Netherlands; 4 Department of Pathology, VU University Medical Center, Amsterdam, the Netherlands; 5 Department of Pathology, AMC University Hospital, Amsterdam, the Netherlands; 6 Molecular Neurogenetics Unit, Department of Neurology, Massachusetts General Hospital and Harvard Medical School, Boston, Massachusetts, United States of America; 7 Department of Pathology, Massachusetts General Hospital and Harvard Medical School, Boston, Massachusetts, United States of America; 8 Department of Neurosurgery, VU University Medical Center, Amsterdam, the Netherlands; Pacific Northwest National Laboratory, United States of America

## Abstract

**Background:**

Glioblastoma multiforme (GBM) cells secrete large amounts of glutamate that can trigger AMPA-type glutamate receptors (AMPARs). This commonly results in Na^+^ and Ca^2+^-permeability and thereby in excitotoxic cell death of the surrounding neurons. Here we investigated how the GBM cells themselves survive in a glutamate-rich environment.

**Methods and Findings:**

*In silico* analysis of published reports shows down-regulation of all ionotropic glutamate receptors in GBM as compared to normal brain. *In vitro*, in all GBM samples tested, mRNA expression of AMPAR subunit GluR1, 2 and 4 was relatively low compared to adult and fetal total brain mRNA and adult cerebellum mRNA. These findings were in line with primary GBM samples, in which protein expression patterns were down-regulated as compared to the normal tissue. Furthermore, mislocalized expression of these receptors was found. Sequence analysis of GluR2 RNA in primary and established GBM cell lines showed that the GluR2 subunit was found to be partly unedited.

**Conclusions:**

Together with the lack of functional effect of AMPAR inhibition by NBQX our results suggest that down-regulation and afunctionality of AMPARs, enable GBM cells to survive in a high glutamate environment without going into excitotoxic cell death themselves. It can be speculated that specific AMPA receptor inhibitors may protect normal neurons against the high glutamate microenvironment of GBM tumors.

## Introduction

High-grade gliomas (glioblastoma multiforme (GBM)) are the most common aggressive malignant brain tumors. Despite new diagnostic techniques and combined modality therapy [1], prognosis remains dismal. GBMs are highly angiogenic and show patterns of invasive growth throughout the brain, making radical surgery impossible and leading in almost all cases to tumor recurrence [2]. Several autocrine and paracrine factors are thought to contribute to the invasive and migratory properties of GBM cells, interacting with the microenvironment and enhancing motility and invasion [3]. Glutamate, one of the major neurotransmitters in the central nervous system, and its metabolism via receptors and transporters, is thought to be a contributing factor to the malignant behavior of GBM [4], [5]. Glutamate binds to both ligand gated ion channels - NMDA (N-methyl-D-apertate), AMPA (α-amino-3-hydroxy-5-methyl-4-isoxazole propionic acid), kainate receptors - and metabotropic glutamate receptors [6]. Intracellular signaling is thought to be initiated upon glutamate-induced modulation of the ion channels in GBM cells [7], [8] as well as in surrounding astrocytes, neurons, pericytes, and endothelial cells [9]–[13]. Specific inhibitors of these receptor channels are therefore thought to exert anti-tumor effects on GBM cells [5].

Down-regulation of astrocytic glutamate transporters and upregulation of the cystine-glutamate antiporter system (system x_c_^−^) in GBM cells results in high concentrations of glutamate in the microenvironment. Glutamate abundance is consequently thought to lead to excitotoxic cell-death of neurons, mainly via the neuronal NMDA receptor, but also via AMPA and kainate receptor overstimulation [9], [10], [14].

Glioma cells are able to regulate calcium conductance by modifying AMPAR subunit-expression (GluR1-4). AMPAR GluR2 subunit transcripts are subjected to RNA Q/R editing, causing receptor impermeability[15]. Interestingly, Labrakakis *et al*. reported that ionotropic GluR activation by glutamate depolarized only a fraction of the GBM cells without showing typical NMDA or non-NMDA currents [16]. Upon modification of GBM cells by viral vector-mediated restoration of the GluR2 subunit, GBM cells failed to form tumors in the brain of nude mice, indicating a pro-survival role for GluR2 down-regulation in GBM [4]. Yoshioka *et al*. described that human neuroblastoma and medulloblastoma cells failed to assemble Ca^2+^-permeable channels, despite expressing a variety of non-NMDA and NMDA receptor genes. This failure confers protection against excitotoxicity and may contribute to progression of tumors of these types [17].

Here, we analyzed AMPA receptor expression and subunit configuration in GBM cells. A decreased ionotropic glutamate receptor, including AMPAR, expression was shown in an *in silico* analysis of primary GBM samples, compared to normal non-neoplastic brain and low-grade gliomas. Our results show down-regulated protein and mRNA expression in GBM cells, from patient material and established cell lines as compared to normal brain cells. We furthermore show partial GluR2 posttranscriptional RNA under-editing in primary brain tumor cells. AMPA receptor blockage with NBQX (2,3-dihydroxy-6-nitro-7-sulfamoyl-benzo[f]quinoxaline-2,3-dione) , a potent competitive AMPA receptor antagonist, conveyed no effect on cell proliferation. These results suggest that down-regulation of functional AMPAR expression is a mechanism allowing GBM cells to escape excitotoxic cell death.

## Results

### *In silico* analysis of AMPAR expression in GBM patients

**Figure 1:**
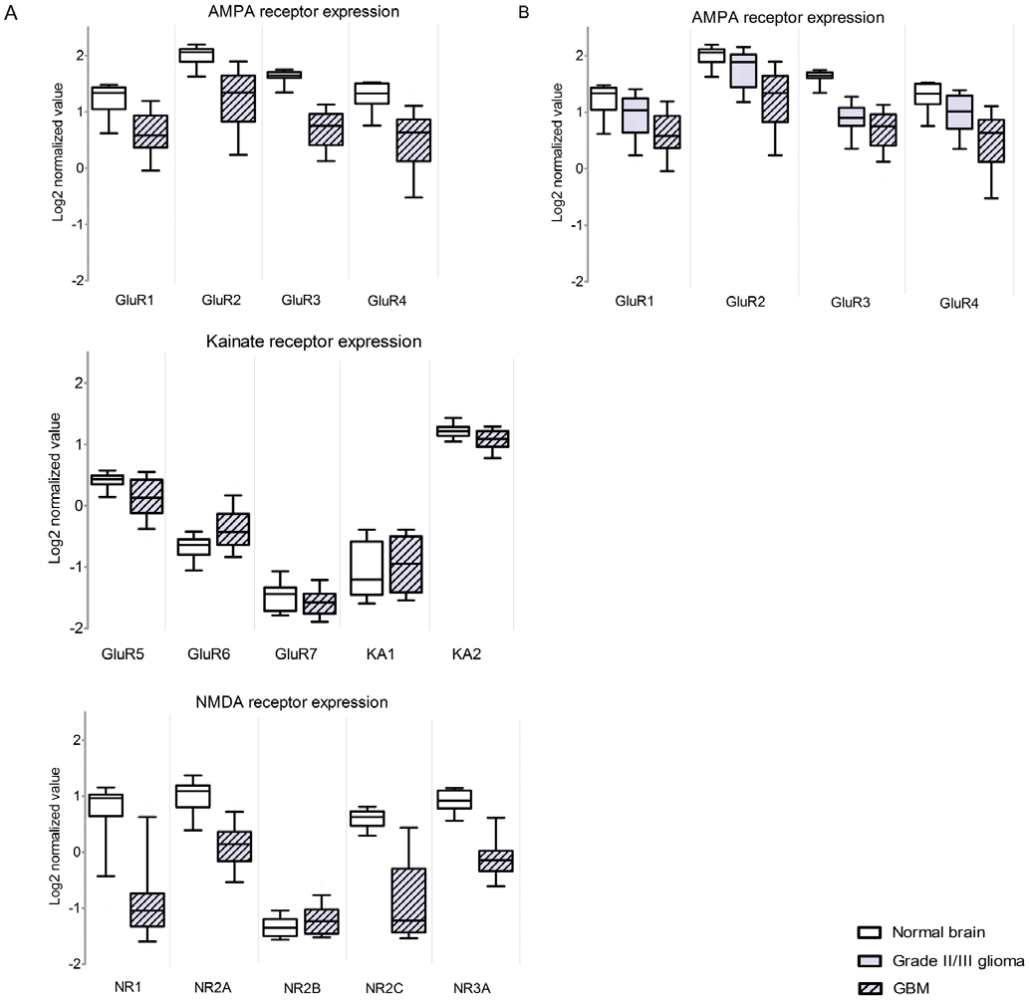
*In silico* analysis of ionotropic glutamate receptor expression levels in GBM tumors. (A) Boxplots of expression of ionotropic glutamate receptors in a dataset of 77 glioblastomas (grey dashed), compared to normal brain from epilepsy surgery (white). p-values and *t*-test values shown in Table S1. (B) Compared to normal brain (white), low grade astrocytoma and oligodendroglioma (n = 76, grey), AMPA receptor (GRIA1–4) expression is decreased in GBM (n = 77, grey dashed) (p<0.0001). Error bars indicate the standard deviation.

In order to determine whether AMPAR subunits are expressed in GBM, we performed *in silico* analysis using Oncomine [18], comparing mRNA expression profiles in dataset of 77 GBMs with 23 samples of non-neoplastic control brain tissue [19] (Figure 1A). An overall down-regulation of ionotropic receptor expression - NMDA (NR1, NR2A–C, NR3A), AMPA (GluR1–4) and kainate (GluR5,7; KA2) receptor - was observed between the controls and the GBM patients, which was statistically significant as calculated by Student's t-test (p<0.0001). No statistically significant difference was observed for GluR7 and KA1. All receptors except for GluR6 showed down-regulation compared to the non-neoplastic control. The largest effect was observed for NMDA receptor NR3A, NR1 and AMPA receptor GluR2, with t-values of 14.665; 10.914 and 8.940, respectively (Table S1). In order to determine whether AMPAR expression correlated with tumor grading AMPAR expression values from low-grade gliomas (WHO grade II oligodendrogliomas and astrocytomas) were compared to the expression values in GBMs (WHO grade IV). AMPA receptor expression was negatively correlated to tumor grading, i.e. oligodendrogliomas and astrocytomas showed higher expression of AMPARs as compared to the GBM samples (Figure 1B). No difference in ionotropic glutamate receptor expression was seen between WHO grade II astrocytomas and oligodendrogliomas (Figure S1).

### Protein expression

To validate AMPAR protein expression in GBM, immunohistochemistry on GluR1 and 2 was performed on 37 paraffin embedded tumor sections of GBM. The multiform phenotype of GBM, with a large variety of different cell types (tumor cells, stem cells, fibroblasts, microglia and endothelial cells), complicates discerning immunostaining of actual tumor cells. A GBM tissue microarray was created with paraffin cores enriched for tumor cells as assessed by two independent neuropathologists. Cell lines of GBM were stained to further substantiate AMPA receptor immunoreactivity in tumor cells.

**Figure 2:**
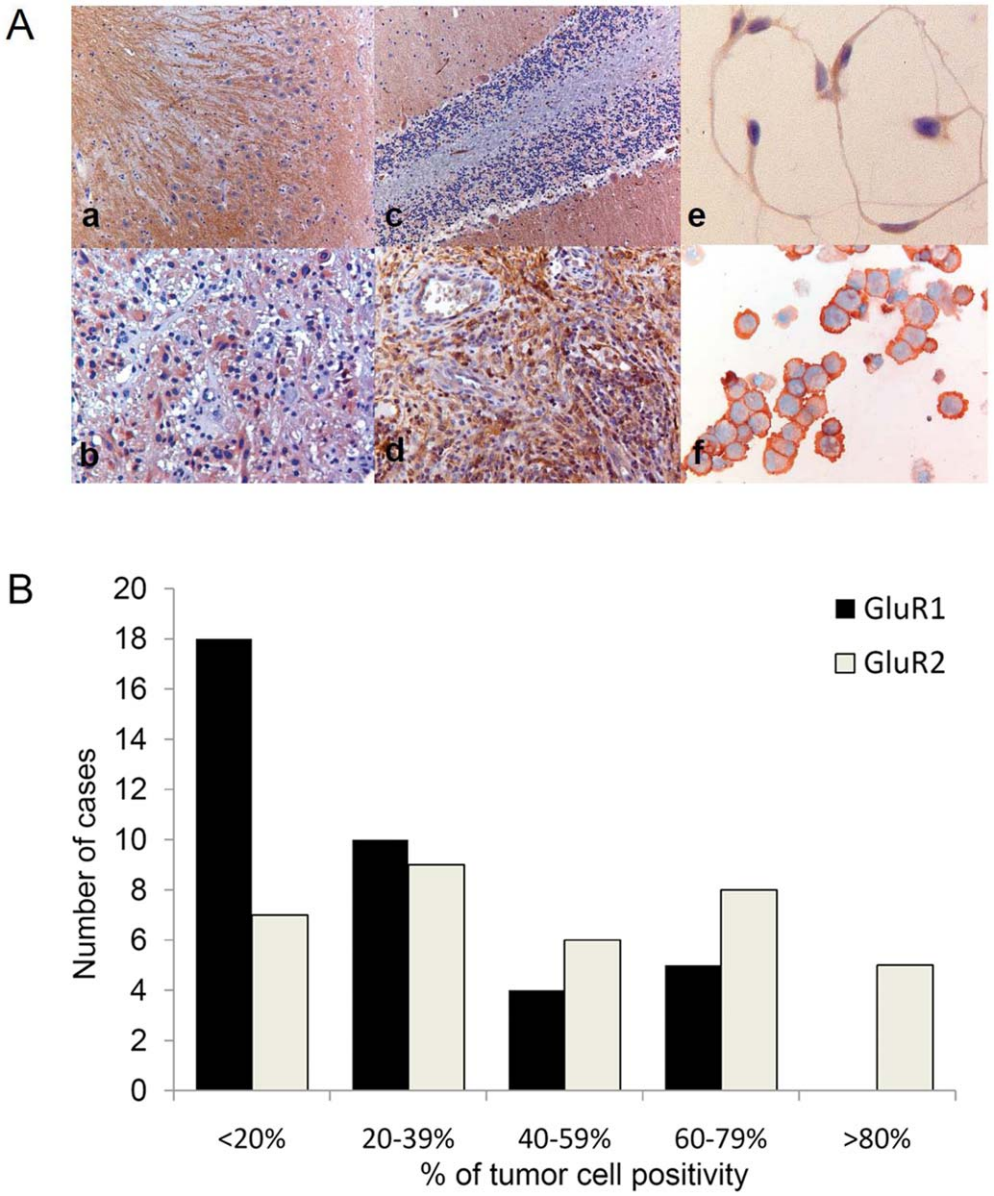
AMPAR immunohistochemistry on paraffin embedded sections. (A) Representative images of (a) GluR1 in hippocampus, (b) GluR1 in GBM, (c) GluR2 in cerebellum, (d) GluR2 in GBM, (e) GluR2 staining in chamberslide of VU-122 cell line, (f) GluR2 staining in cytospin of VU-122. (B) Histogram of protein expression of GluR1 and GluR2 in 37 cases of adult GBM.

No overexpression of AMPARs was observed in these tumor sections, as compared to normal brain tissues cerebellum and hippocampus, in which more abundant and intensive staining was observed (Figure 2A, a–d). Large variation of GluR1 protein expression was observed between patients. Within tumor sections concordance of staining of the different cores allowed for semi-quantitative scoring. Predominantly, cytoplasmatic staining was observed, providing evidence of receptor mislocalization. Only 14% of the cases showed GluR1 expression in more than 60% of the tumor cells (Figure 2B). GluR2 protein expression was also variable between patients and, to a lesser extent within the individual tumor sections, allowing semi-quantitative scoring. GluR2 was mainly localized to the plasma membrane. Expression of GluR2 was higher than GluR1 expression, 36% of the samples showed GluR2 expression in more than 60% of the tumor cells (Figure 2B). In addition, in 9 GBM sections nuclear staining was observed using an antibody directed against GluR2/4 (data not shown).

On cytospin slides of primary VU-122 GBM cells and the U87-MG cell line, membranous staining of GluR2 was observed (Figure 2A, f), whereas in primary VU-028 GBM cells cytoplasmatic GluR2 expression was seen. Chamber slide stainings of VU-028, VU-122 and U87-MG cells also showed GluR2 nuclear and cytoplasmatic localization (Figure 2A, e), again indicative of receptor mislocalization. In summary, GBMs show relatively low but variable GluR1, 2 and 2/4 expression, which is often not localized on the cell membrane.

### GluR mRNA expression and editing in GBM cells

**Figure 3:**
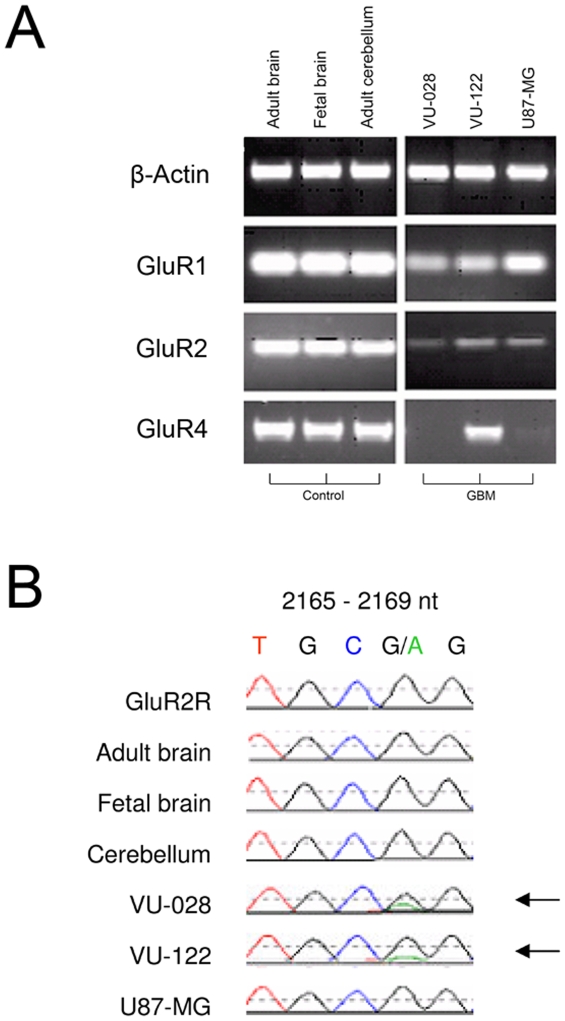
AMPAR mRNA expression in GBM cell lines. (A) RT-PCR of GluR1, 2 and 4 mRNA expression on primary VU-028 and VU-122 GBM cells and the established cell line U87-MG. (B) GluR2 PCR sequencing analysis. Posttransciptional RNA editing at the Q/R site in the second membrane domain (M2) of the GluR2 subunit. The glutamine (Q) codon (CAG) is substituted by an arginine (R) codon (CGG) by replacement of a single nucleotide, adenine to guanine, at position 2168. Adult and fetal brain, adult cerebellum, and glioblastoma cell line U87-MG express fully edited GluR2R (CGG). VU-028 and VU-122 express a combination of both GluR2R and unedited GluR2Q (CAG) subunits.

In order to ascertain mRNA expression, RT-PCR for GluR1, 2 and 4 mRNA expression was performed using 2 primary GBM lines and the established U87-MG GBM cell line (Figure 3A).

None of the cell lines showed mRNA overexpression for these GluR subunits compared to adult and fetal total brain mRNA and adult cerebellum mRNA. The cell lines had a variable expression for GluR1 and 2. In addition, GluR4 expression was measured in all cell lines, except for VU-028 and U87-MG, which showed weak expression (Figure 3A). In order to determine whether GluR2 was edited, the RNA was sequenced of the GBM cells, adult brain, fetal brain and adult cerebellum (Figure 3B). Adult cerebellum, adult brain and fetal brain expressed fully edited GluR2 subunit mRNA, no unedited GluR2Q was detected. Unedited GluR2Q was detected in part of the primary VU-028 and VU-122 GBM cells.

### Role of ion channel functionality in cell proliferation

To further elucidate the role of ion channel functionality, GBM cells were exposed to increasing concentrations of the competitive AMPAR antagonist NBQX and the effect on *in vitro* cell growth was monitored.

**Figure 4:**
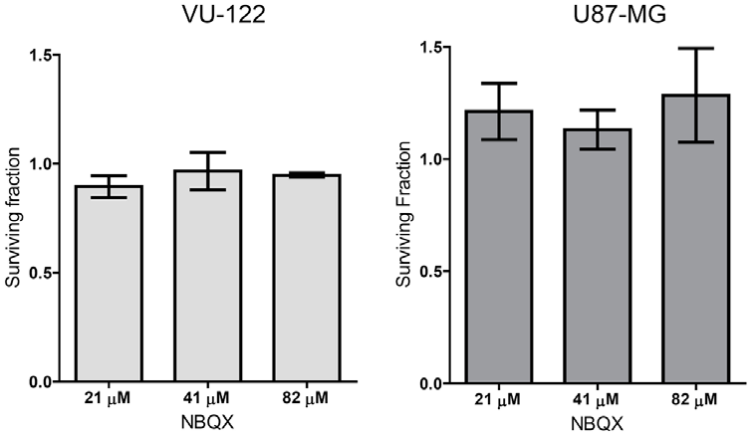
Effect of AMPA receptor antagonist NBQX on proliferation of GBM cell lines. Surviving cells fractions after exposure of GBM cells to different concentrations of the AMPA receptor antagonists NBQX. Shown are the averages of three experiments performed in triplicate. Error bars indicate the standard deviation. Corresponding GluR mRNA subunit expression levels are indicated on the left.

No significant growth inhibition was observed upon AMPAR inhibition, using NBQX concentrations that completely block AMPA and kainate receptors (Ki values of 0.1–0.9 µM and 15.8–19.8 µM, respectively) (Figure 4). These functional experiments indicate that the ion channel function of these AMPAR receptors is not essential for GBM cell proliferation *in vitro*.

## Discussion

Many contradictory results have been published on the relevance of glutamate triggering of AMPAR in GBM progression. Therefore, we investigated how GBM cells survive in a high glutamate environment while the normal surrounding neural cells undergo excitotoxic cell death [9], [10]. We show that down-regulation of AMPAR expression and mislocalization of these receptors enables the cells to survive in this neurotoxic environment. In addition, we found no influence of these receptors in proliferation of the GBM cells, since complete blocking of the electrophysiological function by NBQX did not result in growth inhibition. To substantiate that these receptors may not be functional in GBM we performed several pilot experiments where the glutamate induced currents were measured using whole cell voltage clamped measurements. In all GBM cell lines tested (e.g. VU-028, VU-78, VU-119, VU-121, VU-122, VU-147, VU-154 and U87-MG) we could not detect any proper current response (Figure S2). Due to these negative results in all cells measured so far (>100 cells) at two different laboratories these experiments were not pursued. To fully prove this lack of ion channel function, a more thorough study is warranted including fresh tumor slices. Our results however again suggest lack of AMPAR function in the GBM cells.

Our findings regarding the down-regulated expression of AMPAR are supported by *in silico* analyses that show down-regulated mRNA expression of all ionotropic (NMDA- and non-NMDA) glutamate receptors in primary GBM samples. NMDA receptors showed the highest degree of down-regulation in this dataset, which could provide an additional mechanism of excitotoxicity escape [20].

In summary, in this study we show that on protein level, AMPA-type glutamate receptor subunits are variably expressed in GBM and are overall down-regulated as compared to the normal brain tissue. Besides low and mislocalized expression of AMPA receptors we could not find evidence for ion channel functionality of GBM cells by lack of any depolarization using patch-clamp recordings and lack of growth inhibition after exposure to the AMPAR inhibitor NBQX. These results suggest that stimulation of AMPA receptors - and probably other ionotropic receptors - is not required for GBM cell growth.

Mislocalization of glutamate receptors could be a result of defective trafficking of these receptors. Several proteins are involved in this process. Recently, transmembrane AMPAR regulatory proteins (TARPs) were described to function in specific control of AMPAR and kainate kinetics, ligand affinity and trafficking [21]. Strikingly, *in silico* analysis of TARPs showed strong down-regulation of TARP γ-2 and γ-3 in GBM compared to normal brain [22] (Figure S3), gaining further insight in possible mechanisms of AMPAR mislocalization.

AMPA receptor antagonists were thought to be of potential use as anti-cancer drugs in GBM[5]. Although failing to inhibit GBM cell proliferation, it can be hypothesized that these drugs could still be of potential use in conveying neuroprotection. Using specific inhibitors excitotoxic AMPA and NMDA receptor mediated cell death of neurons could be prevented [23], [24].

Our data provide evidence of down-regulation of AMPAR expression and function in GBM cells and show that these receptors are not essential for the proliferation of these cells. Down-regulation of ionotropic NMDA and non-NMDA glutamate receptors in GBM might allow for the escape of glutamate-mediated toxicity and might facilitate survival in a self-created glutamate rich microenvironment. By imposing excitotoxic cell death on normal neurons and not themselves, GBM cells may manipulate their environment. Based on these findings we speculate that normal neurons might be protected against the high glutamate microenvironment by specific inhibitors of ionotropic receptors [24], [25], but further research is warranted.

## Materials and Methods

### *In silico* analysis

Oncomine [18], a compendium and data-mining application, was used to analyze ionotropic glutamate receptor expression in a transcriptome profile dataset of 77 glioblastoma multiforme samples (WHO grade IV) compared to normal, non-neoplastic brain tissue (n = 23), astrocytoma (WHO grade II) (n = 26) and oligodendroglioma (WHO grade II) (n = 50), as described in the data by Sun *et al.* [19].

### Patient samples

For immunohistochemistry, 37 cases of adult glioblastoma multiforme were included. General written informed consent is obtained from all patients for the use of tumor material. The Cancer Center Amsterdam/VUmc Institute for Cancer and Immunology scientific research committee approved this study. All cases were reviewed independently by two neuropathologists and the diagnosis of GBM was confirmed according to the revised 2007 WHO classification of tumors of the nervous system [26]. A total of 28 cases of GBM were processed onto a tissue micro-array; per patient 3 representative cores with a diameter of 0.6 µm were isolated out of paraffin-embedded tissue. Both paraffin-embedded GBM tissues (n = 9) and the GBM tissue-array were used for immunohistochemistry. As positive controls for immunohistochemistry, post-mortem paraffin embedded tissue of adult hippocampus and cerebellum were used. General written informed consent is obtained from all patients for the use of tumor material.

### Cells and antibodies

Primary cell lines VU-028, VU-078, VU-119, VU-121, VU-122, VU-147 and VU-154 were processed from fresh tumor tissue obtained at surgery from patients with a primary GBM and cultured in Dulbecco's modification of Eagle's medium (DMEM; Flow Laboratories, Scotland) with 10% FCS (Invitrogen), and 1% Glutamine (Invitrogen). Rabbit anti-human GluR1 monoclonal antibody (clone E308) was obtained from Epitomics (Burlingame, CA, USA), anti-Glutamate Receptor 2 monoclonal antibody (MAB397 MsXGluR2, clone 6C4) and anti-glutamate receptor 2 & 4 monoclonal antibody (MAB396) were obtained from Chemicon International (Temecula, CA, USA).

### Immunohistochemistry and -cytochemistry

Tissue sections (5 µm) mounted on superfrost slides (Menzel, Braunschweig, Germany) had undergone dewaxing and rehydration, after which endogenous peroxidase activity was blocked for 30 min in methanol containing 0.3% hydrogen peroxide. Slides were then washed with distilled water and phosphate-buffered saline (PBS; 10 mM, pH 7.4). For antigen retrieval, the slides were placed in sodium citrate buffer (10 mM, pH 6.0) and heated in a microwave oven at 99°C for 10 min, and cooled to room temperature. The sections were washed in PBS and pre-incubated with 10% normal goat serum (NGS) diluted in PBS 30 min prior to incubation with GluR1 antibody (diluted 1∶20) for 2 hr, or GluR2 antibody (diluted 1∶2000) for 1 hr, or GluR2/4 antibody (diluted 1∶200) for 1 hr. The sections were washed with PBS and incubated at room temperature for 1 hr with the appropriate biotinylated secondary antibody diluted in PBS. For the detection of mouse antibodies Power Vision (ImmunoLogic, Duiven, the Netherlands) was used according to the instructions of the manufacturer. Peroxidase activity was detected using 3,3-diaminobenzidine-tetrachloride (Sigma, USA) in 0.1% hydrogen peroxide. All sections were counterstained with haematoxylin and mounted with an aqueous mounting medium (Kaiser's glycerol gelatin, Merck). Immunocytochemistry was performed on cytospins and chamber slides. Cytospins were fixed in icecold buffered formaldehyde-acetone. Chamberslides (Nunc/Thermo Fisher Scientific, Breda, The Netherlands) were fixed using 2% paraformaldehyde. Endogenous peroxidase activity was blocked for 10 min in phosphate-buffered solution (PBS) containing 0.25% hydrogen peroxide. The slides were incubated with the antibodies and peroxidase activity was detected using 3,3-diaminobenzidine-tetrachloride (Sigma, USA) in 0.1% hydrogen peroxide and enhanced with 0.4% cupric sulfate. All sections were counterstained with haematoxylin.

Tissue sections were examined by two independent observers with respect to the presence of neoplastic and/or non-malignant tissue and specific immunoreactivity for the different AMPA receptors of interest. Immunoreactivity was quantified on whole cores of the tissue micro-array (0.6 µm in diameter) and 4 independent fields in whole tissue slides using a light microscope with 25× magnification. Staining indices (number of stained tumor cells per total number of tumor cells) were assigned semi-quantitatively to five categories: <20%, 20–39%, 40–59%, 60–79%, >80%.

### mRNA expression analysis

To semi-quantitatively assess GluR1, GluR2 and GluR4 mRNA expression in glioblastoma cell line (U87-MG) and glioblastoma primary cell lines (VU-028 and VU-122), total RNA was extracted using RNA Bee (Bio-connect BV, Huizen, The Netherlands) according to the manufacturer's instructions. First-strand cDNAs were synthesized with reverse transcriptase by using 1 mg of total RNA as a template and oligo-dT as primers. GluR1, GluR2, GluR4 primer sequences were determined using Oligo 6 software (Molecular Biology Insights, CO, USA). GluR1 (forward), 5′-TTTGAGGAAGGACGGGACCA-3′; GluR1 (reverse), 3′-ATCGTTGGTGGCGTCTGGTG-5′; GluR2 (forward), 5′-TCCTGGTCAGCAGATTTAG-3′; GluR2 (reverse), 3′-ATCGAAAGTGCTGAGGATC-5′; GluR4 (forward), 5′-ACACAGAAGAGCCAGAGGACGGA-3′; GluR4 (reverse), 3′-TGCCCTTGGATTTGCGGACAC-5′; ß-actin primers were used as described [27]. The amplified RT-PCR products were analyzed on 1% agarose gels.

### Sequence analysis of GluR2

Sequence analysis was performed using ABI 3130 system (Applied Biosystems). For sequencing of the GluR2 PCR product, samples were denaturated at 95°C for 3 min, amplified in 45 cycles (1 min of denaturation at 96°C, 15 sec of primer annealing at 55°C, and 4 min of elongation at 60°C).

### Whole-cell voltage clamp recordings

Whole cell voltage clamp recordings were performed as described by Colquhoun *et al.* [28]. Measurements were performed in two independent laboratories. Primary patient GBM cells (VU-028, VU-78, VU-147, VU-154, VU-119, VU-121, VU-122) and commercially available cell line U87-MG) were cultured on glass coverslips. For recordings cells were transferred to a recording chamber containing 126 mM NaCl, 3 mM KCl, 2 mM MgSO_4_, 2 mM CaCl_2_, 10 mM D(+)-glucose, 1.2 mM NaH_2_PO_4_, 26 mM NaHCO_3_ (carboxygenated with 5% CO2/95% O2). Whole-cell recordings were made at room temperature (20–22°C), using 3–4 MΩ borosilicate glass electrodes, containing 77 mM K-gluconate, 77 mM KCl, 0.5 EGTA, 10 mM HEPES, 4 mM Mg-ATP, 4 mM K2Phosphocreatine, 0.4 mM GTP (pH 7.3 with KOH). Cells were voltage clamped at −70 mV, lifted from the glass coverslip, and placed in front of a piezo-controlled (P 245.70, Physik Instrumente, Waldbronn, Germany) fast application system with a double-barreled application pipette. Reliability of application was checked before experiments by measuring the open-tip response to application of diluted (10%) extracellular solution. Current responses were recorded upon 100 ms application of glutamate (1000 µM) using an EPC-8 amplifier with PULSE software (HEKA Elektronik, Lambrecht, Germany). Both primary GBM cells (VU-028, VU-078, VU-121, VU-122, VU-147, VU-157) and established GBM cell line U87-MG were measured several times in this whole cell patch-clamp setup after glutamate application. VU-78 cells were also recorded after AMPA (100 µM) and kainate (100 µM) application. As a control, we tested glutamate application on a nucleated patch pulled from a neocortical pyramidal cell in an acute mouse brain slice.

### Cytotoxicity cell proliferation assay

Proliferation of glioblastoma cell lines VU-122 and U87-MG, growing in a monolayer, were determined using the sulphorhodamine B (SRB) assay. NBQX (1,2,3,4-Tetrahydro-6-nitro-2,3-dioxo-benzo[f]quinoxaline-7-sulfonamide disodium salt; Sigma-Aldrich (St Louis, MO, USA)) was dissolved in H_2_O. Cells were seeded in triplicate at 1×10^4^/ml (VU-122), 4×10^4^/ml (U87-MG) in DMEM with 10% (U87-MG) and 20% FCS (VU-122) in 96-well tissue culture plates (200 µl/well) and incubated with 6 concentrations of NBQX 21–82 µM. After 4 days of incubation the cells were fixed with 30% trichloroacetic acid (TCA) (5 µl/well) at 4°C for 1 hour. After four washes, TCA fixed cells were stained with 0.4% SRB (100 µl/well) for 30 minutes, room temperature. After three washes with 1% ethanoic acid, protein-bound dye was dissolved in 10 mM Tris base (100 µl/well, pH 10.5) and plates were analyzed at 540 nM using a Anthos 2001 plate reader (Anthos-Labtec, Cambridge, UK). Cell doubling time was calculated based on proliferation curves resulting from the change in SRB absorbance over time.

## Supporting Information

Figure S1 In silico analysis of ionotropic glutamate receptor expression levels in low grade astrocytomas and oligodendrogliomas. Boxplots of expression of ionotropic glutamate receptor mRNA in a dataset of low grade gliomas - 50 oligodendrogliomas (white), compared to 26 astrocytomas (grey dashed). None of the GluR gene expression profiles showed major significance in differential expression.
(0.62 MB TIF)

Figure S2 Example traces after application of glutamate to lifted GBM cells. 1000 µM of glutamate was ‘puffed’ during 100 ms to GBM cell lines VU-028, VU-122 and U87-MG. In all cell lines glutamate failed to evoke an inward current, whereas in a pyramidal neuron, glutamate application evoked a large, inward, quickly desensitizing current, mediated by AMPA receptor activation. The trace is the average of 10–20 subsequent applications.
(0.16 MB TIF)

Figure S3 In silico analysis of transmembrane AMPAR regulatory proteins (TARPs) expression levels in GBM tumors. Expression of transmembrane AMPAR regulatory protein gamma 2 and 3 (TARP-γ2 and TARP-γ3) in a dataset of 77 glioblastomas (grey dashed), compared to non-neoplastic, normal brain from epilepsy surgery (white) (p<0.0001, t-test value 8.772 and 12.903 respectively).
(0.25 MB TIF)

Table S1 In silico analysis using Oncomine [18], comparing mRNA expression profiles in dataset of 77 GBMs with 23 samples of non-neoplastic control brain tissue [19]. The t-test value indicating effect size between the two classes, p-value reflecting significance of the differential expression observed.
(0.09 MB RTF)
