# Supplementary figures and images for: Attenuated AMPA Receptor Expression Allows Glioblastoma Cell Survival in Glutamate-Rich Environment
Source: PLoS One. 2009 Jun 18;4(6):e5953. doi: 10.1371/journal.pone.0005953 (PMC2693929; doi:10.1371/journal.pone.0005953)

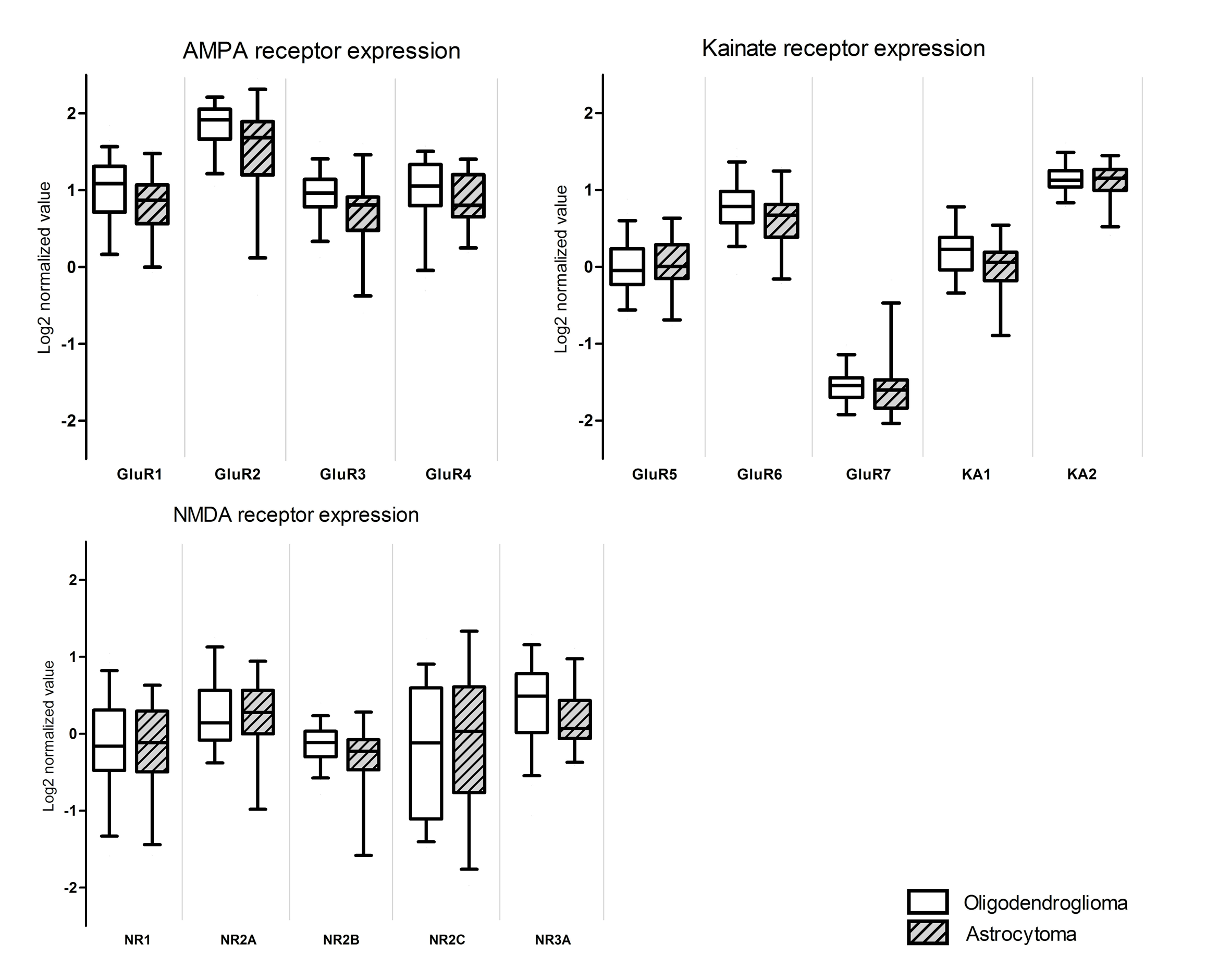

Supplement: Figure S1 — In silico analysis of ionotropic glutamate receptor expression levels in low grade astrocytomas and oligodendrogliomas. Boxplots of expression of ionotropic glutamate receptor mRNA in a dataset of low grade gliomas - 50 oligodendrogliomas (white), compared to 26 astrocytomas (grey dashed). None of the GluR gene expression profiles showed major significance in differential expression. (0.62 MB TIF) [file pone.0005953.s001.tif]

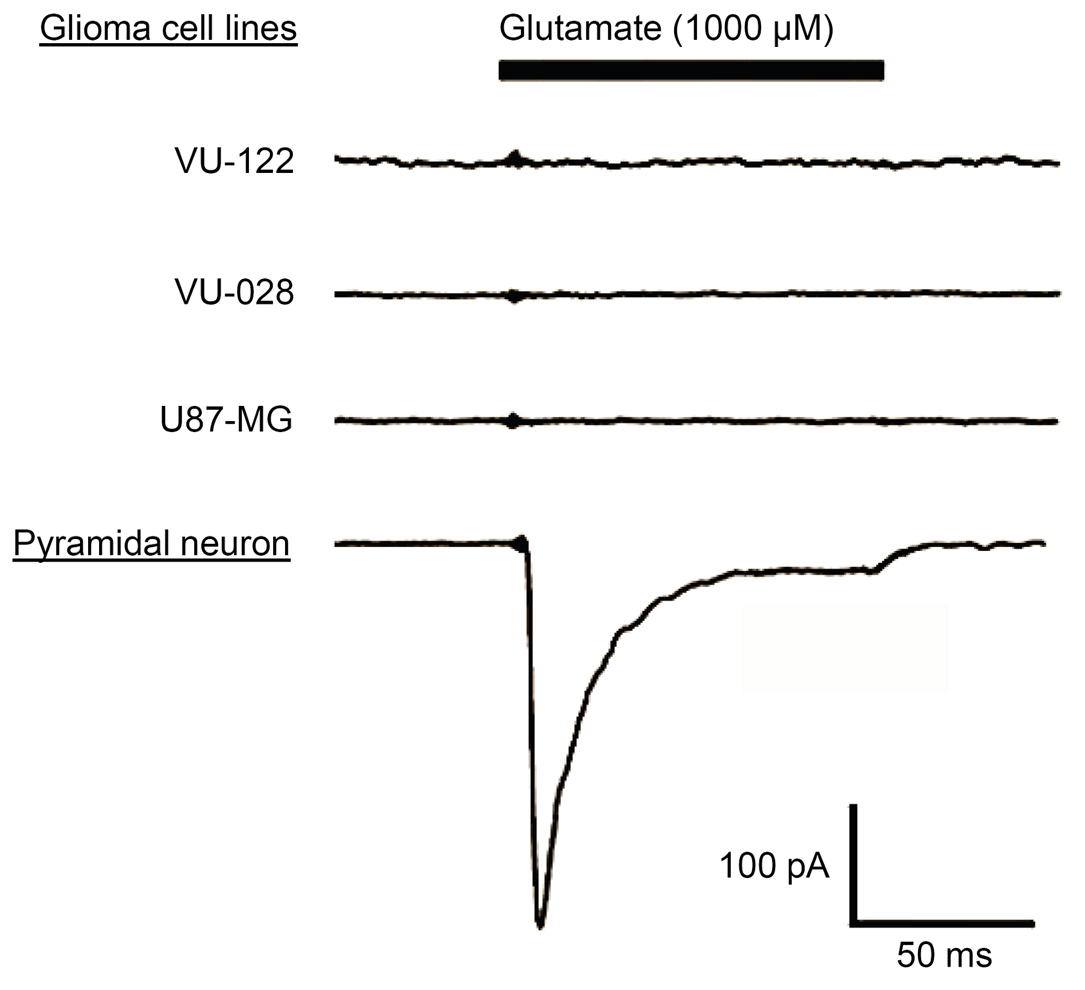

Supplement: Figure S2 — Example traces after application of glutamate to lifted GBM cells. 1000 µM of glutamate was ‘puffed’ during 100 ms to GBM cell lines VU-028, VU-122 and U87-MG. In all cell lines glutamate failed to evoke an inward current, whereas in a pyramidal neuron, glutamate application evoked a large, inward, quickly desensitizing current, mediated by AMPA receptor activation. The trace is the average of 10–20 subsequent applications. (0.16 MB TIF) [file pone.0005953.s002.tif]

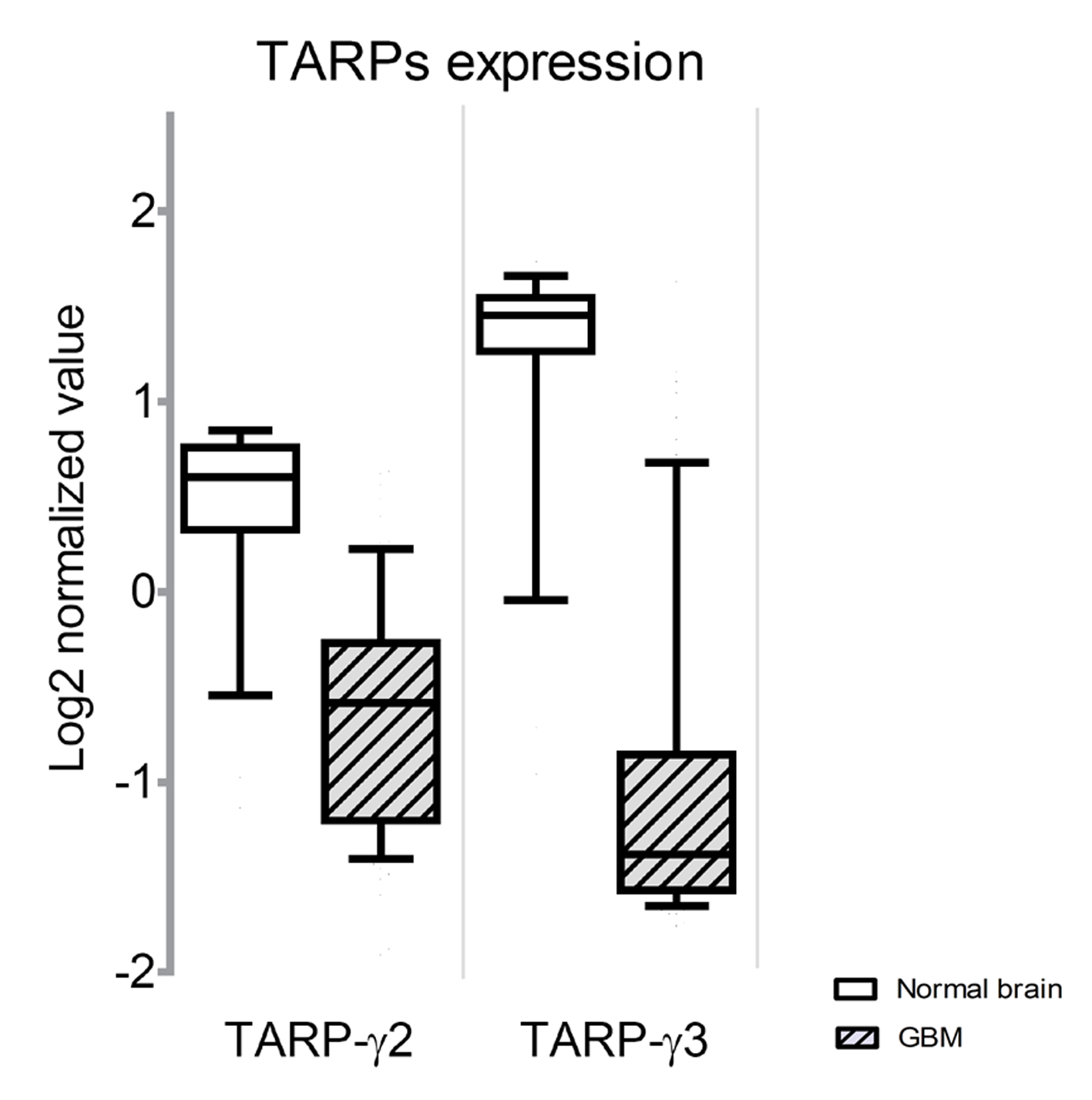

Supplement: Figure S3 — In silico analysis of transmembrane AMPAR regulatory proteins (TARPs) expression levels in GBM tumors. Expression of transmembrane AMPAR regulatory protein gamma 2 and 3 (TARP-γ2 and TARP-γ3) in a dataset of 77 glioblastomas (grey dashed), compared to non-neoplastic, normal brain from epilepsy surgery (white) (p<0.0001, t-test value 8.772 and 12.903 respectively). (0.25 MB TIF) [file pone.0005953.s003.tif]
