# Supplementary material for: Attenuated AMPA Receptor Expression Allows Glioblastoma Cell Survival in Glutamate-Rich Environment
Source: PLoS One. 2009 Jun 18;4(6):e5953. doi: 10.1371/journal.pone.0005953 (PMC2693929; doi:10.1371/journal.pone.0005953)
Supplement: Table S1 — In silico analysis using Oncomine [18], comparing mRNA expression profiles in dataset of 77 GBMs with 23 samples of non-neoplastic control brain tissue [19]. The t-test value indicating effect size between the two classes, p-value reflecting significance of the differential expression observed. (0.09 MB RTF) [file pone.0005953.s004.rtf]

Table S1. Differential mRNA expression of ionotropic glutamate receptor classes.
Receptor 	Gene	Aliases	t-value	p-value	
AMPA	GRIA1	GLUH1, GluR1, GLURA, HBGR1, MGC133252	6.348	< 0.0001	
	GRIA2	GluR-K2, GluR2, GLURB, HBGR2	8.940	< 0.0001	
	GRIA3	GluR-C, GLUR-K3, GluR3, GLURC	6.496	< 0.0001	
	GRIA4	GluR4, GLUR4C, GLURD	8.375	< 0.0001	
	
Kainate	GRIK1	EAA3, EEA3, GLR5, GluR5	5.275	< 0.0001	
	GRIK2	EAA4, GLR6, GluR6, MGC74427, MRT6	- 4.889	< 0.0001	
	GRIK3	EAA5, GLR7, GluR7, GluR7a	1.064	0.293	
	GRIK4	EAA1, GRIK, KA1	- 0.948	0.349	
	GRIK5	EAA2, GRIK2, KA2	4.260	< 0.0001	
	
NMDA	GRIN1	NMDA1, NMDAR1, NR1	10.914	< 0.0001	
	GRIN2A	NMDAR2A, NR2A	8.307	< 0.0001	
	GRIN2B	hNR3, MGC142178, MGC142180, 
NMDAR2B, NR2B	-2.310	0.024	
	GRIN2C	NMDAR2C, NR2C	2.324	< 0.0001	
	GRIN3A	FLJ45414, NMDAR-L, NR3A	14.665	< 0.0001	

In silico analysis using Oncomine [18], comparing mRNA expression profiles in dataset  of 77 GBMs with 23 samples of non-neoplastic control brain tissue [19]. The t-test value indicating effect size between the two classes, p-value reflecting significance of the differential expression observed. 
